# Development of a Mixed-Reality Application for Volitional Step Training in People With Multiple Sclerosis: Cross-Sectional Qualitative User-Centered Co-Design Study

**DOI:** 10.2196/94312

**Published:** 2026-09-14

**Authors:** Gabriele Perachiotti, Isolde Martina Busch, Ersilia Vallefuoco, Giovanni D'Errico, Nicola Moccaldi, Sofia Straudi, Pasquale Arpaia

**Affiliations:** 1 Department of Neurosciences and Rehabilitation University of Ferrara Ferrara, Emilia-Romagna Italy; 2 Department of Neurosciences Arcispedale Sant'Anna Ferrara, Emilia-Romagna Italy; 3 Department of Neurosciences, Biomedicine and Movement Sciences University of Verona Verona, Veneto Italy; 4 Department of Electrical Engineering and Information Technology University of Naples Federico II Napoli, Campania Italy; 5 Department of Cultural Heritage University of Salento Lecce, Puglia Italy

**Keywords:** falls, mixed reality, multiple sclerosis, postural balance, serious games, user-centered design

## Abstract

**Background:**

People with multiple sclerosis (MS) are at high risk of falls, with approximately half experiencing at least 1 fall within 6 months. Multidirectional step training has shown potential for fall prevention, but evidence in people with MS remains inconsistent. Mixed reality (MR) offers adaptive and home-based rehabilitation opportunities. However, successful implementation of MR-based serious games requires structured co-design processes integrating patient, clinician, and technical perspectives.

**Objective:**

This qualitative study describes the co-design and prototyping of a home-based MR serious game called Head Your Limits for volitional step training in people with MS, aiming to identify patient-, clinician-, and technology-driven design requirements and translate them into an MR rehabilitation application.

**Methods:**

We implemented a cross-sectional qualitative user-centered co-design process. Phase 1 involved 2 online focus groups with people with MS to identify experiential and design preferences related to MR-based training. Phase 2 included a focus group with 4 clinicians and 1 engineer to define clinical and technical requirements. Phase 3 consisted of in-person hands-on testing of 2 MR prototypes (“clock” and “grapes”), developed in Unity and deployed on Meta Quest 3. Twenty people with MS were purposively screened from among individuals referred for motor rehabilitation; 14 participated in the online focus groups and 9 in in-person prototype testing (mean age 56.2, SD 7.0 years; mean Expanded Disability Status Scale score 5.1, SD 1.3; 6/9, 66.7% female). Iterative refinement followed each phase, translating stakeholder-derived requirements into architectural modifications of the MR application. Focus group recordings and moderator notes were reviewed to identify themes and design requirements guiding iterative prototype refinement.

**Results:**

Five primary patient-derived requirements emerged: multisensory feedback, gamification, progress tracking, visual accessibility, and ergonomic comfort. Clinicians emphasized adaptive difficulty, cognitive-motor integration, biomechanical validity, and caregiver compatibility. Prototype comparison showed that floor-level targets induced cervical discomfort and postural compensation, whereas auditory guidance reduced head flexion. The final application repositioned targets at eye level and implemented head-mounted display tracking to ensure appropriate center-of-mass displacement, preserve proprioceptive engagement, enable caregiver proximity, and integrate gamified feedback and performance reporting.

**Conclusions:**

Iterative stakeholder engagement resolved key design tensions related to ergonomics, sensory reliance, biomechanical validity, and motivation. The transition to eye-level, head-mediated interaction emerged as a core solution integrating therapeutic and user-experience goals. Through a rigorous user-centered co-design process, aligned with contemporary human-centered development models, we developed an MR serious game for volitional step training in people with MS. This study contributes to the field by providing a transparent methodological framework integrating biomechanical validity, usability, and engagement. Although clinical effectiveness remains to be tested, these findings may inform the design of accessible, personalized home-based rehabilitation for people with MS and other neurological populations.

## Introduction

### Description of Research Problem

Multiple sclerosis (MS) is a complex neurological disorder that leads to cognitive and motor deficits [1]. Cognitive deficits affect up to 60% of people with MS, limiting their activities of daily living and capacity for work [2]. The most prevalent cognitive dysfunctions are slowing of information processing speed and impairments in episodic memory, attention, and executive functions [3]. Motor dysfunction is a highly disabling feature of MS, resulting from the multifocal involvement of the central nervous system. Damage to motor, sensory, cerebellar, and brainstem pathways leads to impairments in strength, coordination, balance, and gait, which often coexist and interact, producing highly variable motor phenotypes across individuals [4,5]. These motor deficits significantly impact functional independence and quality of life and represent one of the main drivers of disability progression in people with MS [6]. Importantly, the expression of motor dysfunction in MS is not static but evolves over time, reflecting disease course, lesion distribution, and the deployment of compensatory mechanisms [7]. Consistent with this multifactorial impairment, cognitive and motor disorders are strongly associated with an increased risk of falling [8,9]. Indeed, at least 50% of people with MS experience 1 or more falls within a period of 3 to 6 months, with a large proportion sustaining concomitant injuries ranging from mild trauma to injuries requiring hospitalization [10]. Beyond the immediate physical consequences, falls often lead to activity restriction, which in turn contributes to reduced social participation and decreased levels of physical activity, further exacerbating disability and negatively impacting quality of life [10,11].

Several approaches have been proposed to prevent falls in people with MS, including balance rehabilitation, combined motor and sensory training, mind-body exercises, functional electrical stimulation, and education-based programs [12]. However, although there is strong evidence that these interventions can improve balance in people with MS, clear effectiveness in reducing falls has not yet been demonstrated [12]. In the absence of clear evidence on how to effectively reduce falls in people with MS, insights from fall-prevention research in older adults may provide important indications. In this population, the systematic review by Sherrington et al [13] recommends engaging in balance-challenging exercise for at least 2 hours per week for a minimum of 6 months to achieve meaningful reductions in falls [13]. Beyond the training dose, emerging evidence suggests that the content of balance training is also critical. A systematic review by Okubo et al [14] demonstrated that multidirectional step training can prevent falls by approximately 50%.

In this context, there is a clear need for rehabilitation interventions that can be safely performed at home, without continuous direct supervision by a physiotherapist, to achieve the high training volumes required for effective fall prevention [13]. Furthermore, these interventions should be sufficiently task-specific, challenging, and engaging to be effective and to promote long-term adherence [14]. In response to these requirements, volitional multidirectional step training interventions have been proposed for people with MS [15-17], with the aim of training stepping responses, which represent the final opportunity to recover stability following a perturbation [18]. Hoang et al [16] developed a large-scale stepping-based training program for people with MS. Despite a large sample size and an extended intervention duration, this program did not demonstrate a reduction in fall rates. A potential limitation may be the spatial organization of the stepping task, which is constrained by the stepping mat and limited to 4 predefined directions. Such a spatial constraint may reduce the ecological validity of the task, as balance recovery during everyday tasks requires steps in all directions. In this regard, task specificity has been identified as a critical factor in reactive balance training [19].

Among technological solutions, emerging extended reality (XR) technologies offer new approaches for people with MS, especially in motor rehabilitation [20]. XR technologies, which include virtual reality, mixed reality (MR), and augmented reality, combine virtual and physical worlds to provide immersive, realistic experiences in which users can practice specific skills or behaviors [20,21]. Several studies have shown that the sense of presence and immersion elicited by XR environments may influence performance and rehabilitation outcomes in people with MS. XR systems also facilitate the replication of task-oriented activities related to daily living, ensuring ecological validity [22]. XR-based applications often incorporate serious game (SG) frameworks to enhance patient engagement and motivation, particularly during prolonged or repetitive training [22]. Furthermore, given the challenges that people with MS face in adhering to rehabilitation programs due to personal, social, and economic factors, XR-based SGs can help reduce barriers to accessing rehabilitation programs [23]. Another important feature is the ability to customize extended environments and design applications that adapt in real time to the patient's performance [24]. The ability to create enriched and highly adaptable environments, tailored to individual postural control abilities [25], is particularly important in MS, as motor phenotypes are highly variable, reflecting differences in lesion location, disease course, and compensatory mechanisms [26]. Second, XR can provide rich audiovisual cues [25] and closed-loop feedback [27], both of which are pivotal for promoting motor learning [28]. Motor learning is the mechanism underlying the production of more effective movement [28], and it is preserved even in people with MS with higher levels of disability [29]. Among XR environments, MR has been shown to elicit higher levels of spatial presence [30]. Spatial presence is critical for the execution of confident body movements [30]. This increased confidence in movement may encourage people with MS to explore the limits of their motor abilities within the MR environment, which is critical for driving functional improvements [31]. Spatial presence is also an essential element of embodiment [32], a process that is commonly disrupted in people with MS when interacting with virtual environments [33].

While MR offers significant technological potential for motor rehabilitation in MS, its successful translation into clinical practice depends on development strategies that effectively integrate the perspectives of end users and other key stakeholders [20]. Co-design methods are increasingly acknowledged as essential in the development of health care technologies, especially when multiple stakeholder perspectives, including patients, clinicians, and engineers, must be integrated [34,35]. In the context of MR-based rehabilitation devices for individuals with MS, a co-design framework ensures that experiential, clinical, and technical requirements inform all stages of the design process, in line with biopsychosocial models of care and patient-centered innovation [35,36]. Various co-design approaches are available, including participatory design, cooperative inquiry, and living lab methods, which differ in stakeholder composition, duration, and tools [37-39]. The selection of a specific approach should reflect the number and diversity of stakeholders involved, the complexity of the technology, regulatory and safety constraints, and the development timeline, as these factors critically influence the real-world adoption and sustained use of rehabilitation technologies [34,40,41].

Among the available co-design tools, focus groups are particularly well suited to the development of complex health care technologies that require the integration of experiential, clinical, and technical perspectives [35,42]. Focus groups facilitate interactive discussions in which stakeholders can articulate needs, negotiate priorities, and collectively surface design ideas [43,44]. Unlike individual interviews or survey-based approaches, they enable the emergence of themes shaped by group dynamics, such as trade-offs between clinical safety parameters and usability, or between technical feasibility and patient motivation [42,45]. This dialogic format is especially appropriate for harmonizing the perspectives of patients, who may experience fatigue, sensory discomfort, or motivational challenges, with those of clinicians, who prioritize therapeutic efficacy and safety, and engineers, who focus on system performance, latency, motion tracking, and real-time feedback [34,35]. The iterative use of focus groups further supports the progressive refinement of design assumptions and the exploration of usability and acceptability across multiple development stages, consistent with human-centered and participatory design frameworks [36,40]. In health care co-design, focus groups are frequently used in early design phases because they support rapid sense-making across stakeholder groups and enable the identification of tacit assumptions that may not emerge through individual methods. Their capacity to approximate real-world decision-making processes makes them particularly relevant for the initial development of medical devices and digital health interventions [34,45].

### Research Goals

The aim of this qualitative study was to describe the user-centered co-design, iterative prototyping, and conceptual development of a home-based MR SG for volitional step training in people with MS. Specifically, we aimed to identify patient-, clinician-, and technology-driven design requirements and translate these into an MR rehabilitation application integrating therapeutic relevance, usability, safety, and engagement. The application was designed for patients not requiring bilateral support to walk. The development of SGs for rehabilitation benefits from a structured, human-centered methodology that ensures usability, acceptability, and alignment with patients’ needs. In this work, the ideation and refinement of our MR-based motor training application were inspired by the International Organization for Standardization (ISO) 9241-210 framework for human-centered design of interactive systems [46]. The standard outlines the following six core principles: (1) explicit understanding of users, tasks, and context of use; (2) user involvement throughout the design and development process; (3) iterative refinement driven by user-centered evaluation; (4) design responding to user needs and requirements; (5) consideration of the whole user experience; and (6) multidisciplinary design teams that guide the structure and logic of the co-design pathway. These principles guided the structure and logic of the co-design pathway.

## Methods

### Research Design Overview

This study used a cross-sectional qualitative, user-centered co-design approach involving iterative prototype development. The methodology was operationalized through a 3-phase pipeline using focus groups with distinct stakeholder categories at each stage, with the overall aim of informing the design, refinement, and evaluation of the MR application for volitional step training.

Phase 1 aimed to explore the perspectives of individuals with MS regarding their rehabilitation experiences, expectations, preferences, and perceived acceptability of MR-based training. The focus groups were designed to elicit participants’ views on practical aspects of use, including comfort, fatigue, and preferred feedback modalities, as well as broader considerations related to motivation and engagement.

Phase 2 aimed to gather interdisciplinary input from health care professionals and an engineer to contextualize patient perspectives within clinical and technical constraints. This phase focused on discussing clinical applicability, safety considerations, and feasibility aspects relevant to the development of the MR application.

Phase 3 aimed to evaluate user interaction with the MR application through hands-on testing with individuals with MS. This phase was designed to collect both quantitative and qualitative feedback on usability, user experience, perceived usefulness, motivation, and intention for future use, in order to support iterative refinement of the system.

### Study Participants

#### Researcher Description

Each focus group was moderated by a psychologist and a physiotherapist with previous research experience in MS rehabilitation. Their understanding of balance training, rehabilitation technologies, motivation, and human-technology interaction informed the development of the focus group structure. The broader research team was multidisciplinary and included a rehabilitation physician with extensive experience in MS rehabilitation and translation of rehabilitation technologies into clinical practice (SS), as well as engineers with expertise in digital health technologies, XR systems, and human-computer interaction for health care applications. The different backgrounds allowed the individual disciplinary assumptions to be balanced and ensured that decisions regarding prototype refinement were guided by stakeholder feedback rather than by the prior expectations of any single researcher.

#### Researcher-Participant Relationship

The psychologist had no prior therapeutic relationship with the participants and therefore acted as the primary moderator of the focus groups to ensure independent facilitation of the discussions. The physiotherapist had an existing therapeutic relationship with the participants, as all had previously received care at the rehabilitation unit. His role was primarily supportive, using the established rapport to facilitate open communication and encourage participants to share their experiences. The other members of the research team had no relationship with the participants.

#### Participant Recruitment

For the patients’ focus groups, people with MS were recruited among individuals who had been referred to the Ferrara University Hospital for motor rehabilitation in the previous year. Eligible participants were contacted by telephone and invited to participate. For the health care professionals and engineer focus group, colleagues and external collaborators working in the field of rehabilitation technology and with expertise in neurological rehabilitation were invited via email.

#### Participant Selection

People with MS were selected using purposive sampling principles [47] with the aim of including people with MS presenting different levels of disability and, therefore, potentially different needs, movement constraints, and perspectives relevant to MR balance training design. To ensure heterogeneity of experience, participants in phase 1 were intentionally recruited across a range of disability levels. Two separate online focus groups were conducted: the first included participants with higher disability levels, while the second included participants with lower disability levels.

In line with the qualitative and co-design nature of the study, no formal statistical sample size calculation was performed, and recruitment was not guided by a predefined thematic saturation threshold. Instead, participant involvement was guided by the iterative needs of the prototype development process. After each focus group, our research group pragmatically assessed whether the discussion had generated clear, recurrent, and actionable design-relevant information to inform the next co-design phase, including decisions about prototype features, safety requirements, usability issues, and technical feasibility.

People with MS were eligible if they met the following inclusion criteria: aged 18 to 75 years, diagnosis of MS, disability level lower than 6.5 as assessed using the Kurtzke Expanded Disability Status Scale (EDSS) [48], adequate digital skills, and the presence of a caregiver. Participants were excluded if they required bilateral support to walk or had a Mini-Mental State Examination (MMSE) score lower than 24, consistent with the conventional cutoff used to identify individuals with cognitive impairment [49].

#### Participant or Other Data Sources

A total of 20 people with MS were initially screened for participation (Figure 1). Of these, 6 were eligible but did not participate due to logistical constraints. Therefore, 14 people with MS were enrolled in the online focus groups, including 5 individuals with an EDSS score greater than 5.5, another 5 with EDSS scores between 4 and 5.5, and 4 with EDSS scores less than 4. Of these 14 participants, 9 subsequently attended the in-person focus group, which included hands-on interaction with the prototype demonstrations. The remaining 5 participants were unable to take part in the testing phase due to logistical constraints.

The mean age of the in-person focus group was 56.2 (SD 7.0) years, with a mean EDSS score of 5.1 (SD 1.3) and a mean disease duration of 12.0 (SD 6.3) years. Six participants (66.7%) were female. Most participants required a walking aid, with 6 (66.7%) using a single cane and 3 (33.3%) walking without support. Both relapsing-remitting and progressive MS phenotypes were represented in the sample.

The clinician and engineer focus group included 3 physiotherapist researchers, 1 physiatrist researcher, and 1 biomedical engineer. All participants had expertise in MS and MS-related interventions.

**Figure 1:**
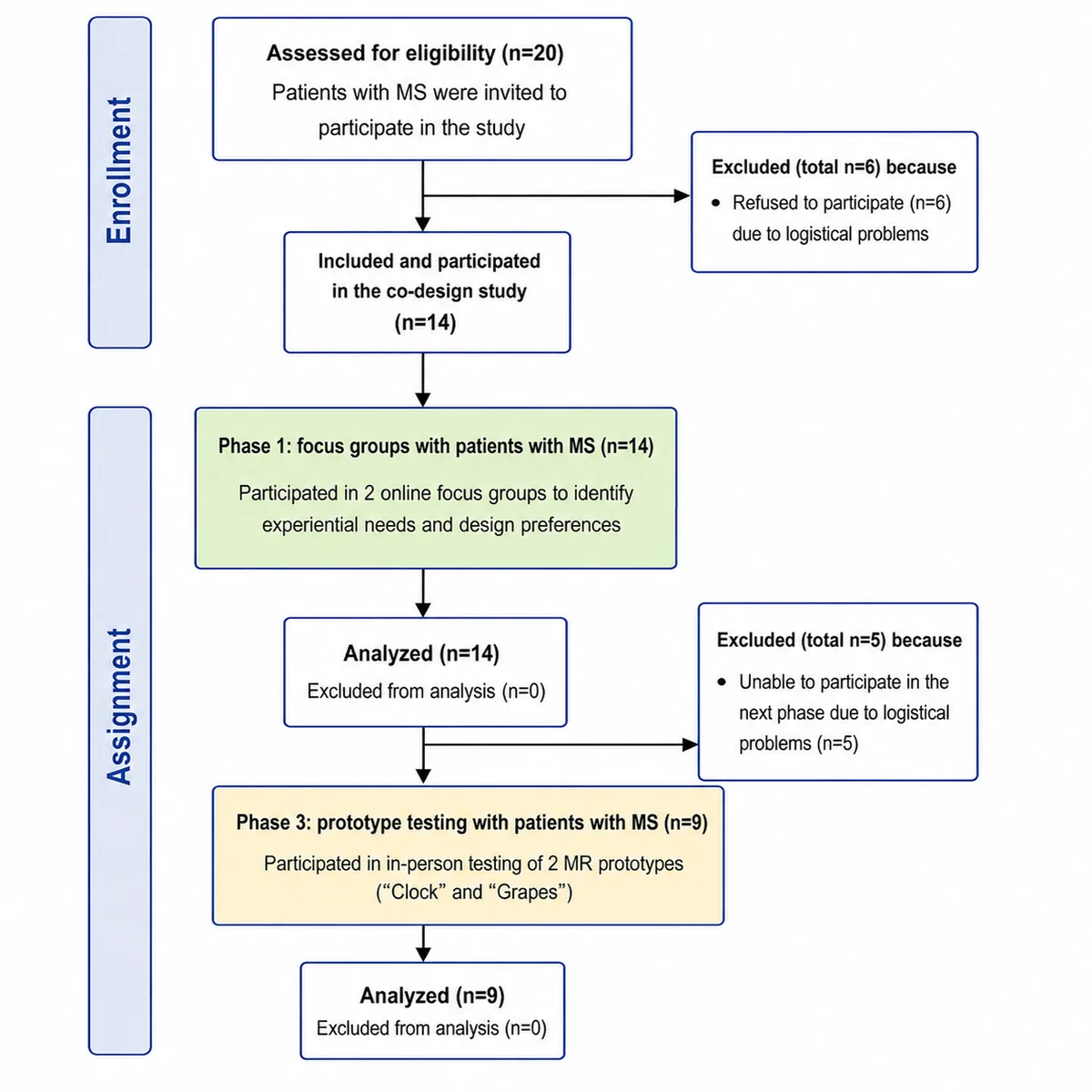
Participant flow through the co-design study. Flow diagram showing participant recruitment, inclusion, exclusion, and progression through phase 1 focus groups and phase 3 in-person prototype testing. Twenty people with multiple sclerosis were assessed for eligibility; 14 participated in phase 1, and 9 completed phase 3. A total of 6 declined participation at enrollment, and 5 were unable to participate in phase 3 because of logistical reasons. MR: mixed reality.

### Data Collection

#### Design and Procedure

All focus groups were conducted using a semistructured format and moderated by 2 trained researchers. Discussions followed ad hoc discussion guides while allowing flexibility to explore emerging topics raised by participants.

The 2 initial patient focus groups (taking place on the same day) and 1 interdisciplinary focus group were conducted remotely via Zoom (Zoom Communications, Inc), whereas the final patient focus group was conducted in person. The interdisciplinary focus group, involving health care professionals (n=4) and 1 engineer, was conducted using a parallel semistructured format. All focus groups conducted via Zoom were video recorded.

The third patient focus group was conducted in person and centered on hands-on testing of the MR applications. Following hands-on testing of both prototypes, participants completed the System Usability Scale (SUS) [50] as an exploratory quantitative measure of overall perceived usability of the MR experience [51]. This session also included a brief focus group discussion, which was audio recorded.

Semistructured discussion guides were developed for both the initial patient focus groups and the interdisciplinary focus group. The full outlines and guiding questions are reported in Multimedia Appendices 1-3. The focus group guides were developed through discussion within the multidisciplinary research team, which included clinical, psychological, rehabilitation, and technological expertise. This process was intended to ensure that the guides addressed both the experiential perspectives of end users and the clinical and technical requirements relevant to the development of the MR application. The content of the guides was aligned with the ISO 9241-210 principles for human-centered design, established focus group methodology [42,43,45], and the specific objectives of each co-design phase.

#### Recording and Data Transformation

The findings emerging from each phase of the co-design process were systematically documented and used to inform subsequent stages. Following each focus group, the moderators produced structured summary reports synthesizing key discussion points and design-relevant considerations. These reports did not aim to provide exhaustive qualitative analyses, but rather to capture actionable insights relevant to the iterative development of the MR application. Outputs from the initial patient focus groups were first shared within our research team and subsequently discussed during the interdisciplinary focus group with experts. In this focus group, patient-derived perspectives were reviewed to support shared interpretation and prioritization of design-relevant insights. Similarly, insights emerging from the interdisciplinary focus group were summarized and communicated back to our research team and used to guide the preparation of the MR application prototypes evaluated in the subsequent patient focus group. During the final patient session, selected elements derived from earlier phases were explicitly presented to participants to support informed discussion and hands-on evaluation.

#### Data-Analytic Strategies: Review and Synthesis of Focus Group Material

After each session, focus group recordings and moderator notes were reviewed using a pragmatic descriptive approach. The review focused on identifying recurrent discussion points, user preferences, perceived barriers, safety and usability concerns, and design-relevant requirements that could inform subsequent prototype refinement. Particular attention was given to comments related to acceptability, comfort, feedback modalities, motivational elements, accessibility, home-based use, clinical applicability, and technical feasibility. Based on this review, the moderators prepared structured summary reports capturing the main design-relevant considerations emerging from each focus group. These reports were used to organize the focus group material into actionable co-design outputs and to document the main considerations informing iterative prototype refinement. The analytic process was therefore oriented toward the generation of practical design insights rather than toward formal thematic analysis, while ensuring transparent reporting of the data sources and procedures used to summarize and interpret the focus group material.

#### Methodological Integrity

Methodological integrity was supported through the iterative and multidisciplinary nature of the co-design process. The inclusion of patients with different disability levels, together with clinicians and engineers, ensured that multiple perspectives informed the development of the application. Throughout the study, stakeholder feedback was systematically documented, discussed within the multidisciplinary team, and translated into successive prototype refinements. Findings are supported by representative participant quotations and by a transparent description of how qualitative insights informed specific design decisions, thereby enhancing the credibility and practical utility of the study.

#### Technological Tools

Based on the results of the first 2 focus group sessions, 2 demos were developed. The first demo, namely, the clock demo, simulated a virtual clock and required users to select the correct number displayed in the environment. The target number was indicated through an artificial voice, created via ElevenLabs (Eleven Labs Inc). When the correct button was pressed, the system provided both visual feedback (the selected number turned green) and auditory feedback (Figure 2). All buttons remained visible on the floor throughout the task.

The second demo, named grapes (Figure 3), required players to interact with a bunch of grapes that appeared at randomly selected clock-face positions in the environment, one at a time. Each time a bunch appeared, the player was required to press it (selection is performed by stepping onto the desired floor button; contact is detected via collision logic and confirmed by visual and auditory feedback). Upon interaction, the system provided visual and auditory feedback, represented by the visual explosion of the grape bunch accompanied by a sound effect.

Both demos were developed in Unity (version 2022.3.45f1) and deployed for Meta Quest 3/3S. The applications were kept deliberately basic in order to facilitate a clearer, more focused, and more reliable co-design evaluation process. The head-mounted display (HMD) was selected based on device characteristics associated with improved comfort and usability in immersive applications, including high resolution, low latency, and relatively low weight, which have been reported among the hardware-related factors influencing cybersickness [52]. A key technological challenge concerned the detection of foot movements without relying on external sensors beyond the MR headset. A key limitation of current MR systems is their use of inside-out tracking architectures, which are mainly focused on head pose estimation and, in some cases, hand or controller tracking. On the other hand, using external sensors limits the feasibility of all-in-one solutions suitable for all rehabilitation settings. To address this issue, 2 different approaches were explored. In the first application (clock demo), the default Meta Blocks tools were used to define the player, track their position within the mixed environment, and manage collisions with the virtual clock elements. In the second demo (grapes demo), player interaction with the grape bunches was instead detected through the occlusion system, enabling collision-based interaction directly within the MR space. Specifically, foot-target interaction was inferred by combining Meta’s environment depth-based occlusion rendering with custom collision detection logic implemented in C#. A volumetric probe was defined above each target and periodically queried using the MR Utility Kit Environment Raycast Manager to detect the presence of real-world geometry within the target space. To reduce false positives due to depth noise, a temporal threshold was applied before confirming a valid interaction event, which then triggered spatialized audio and visual feedback.

**Figure 2:**
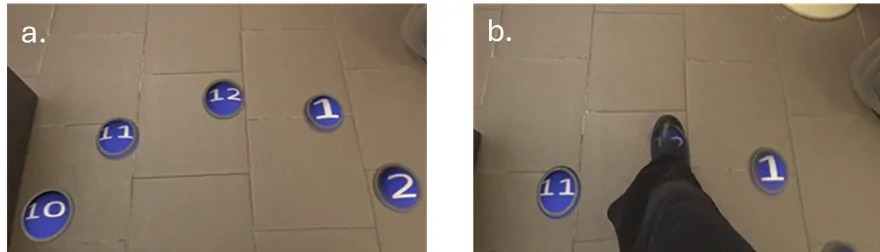
Clock demo. The figure illustrates the clock prototype developed as part of the user-centered co-design process. (A) Participants wearing a Meta Quest 3 (Meta Platforms, Inc) head-mounted display were prompted by an auditory cue to step on a specific number positioned within a virtual clock layout to promote multidirectional stepping and weight shifting. (B) Successful task completion triggered visual and auditory feedback to reinforce performance and engagement.

**Figure 3:**
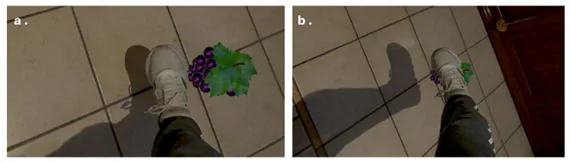
Grapes mixed reality (MR) demo. The figure illustrates the grapes MR prototype developed as part of the user-centered co-design process. (A) The virtual grape bunch appears on the floor in an unpredictable position as a stepping target to promote multidirectional stepping and lower-limb weight shifting within the MR environment. (B) Interaction is triggered when the user’s foot overlaps the target area, progressively occluding the virtual object through depth-based occlusion, and is confirmed through visual and auditory feedback.

### Ethical Considerations

The study was approved by the Ethics Committee Area Vasta Emilia Centro (AVEC; approval number 422/2025/Disp/AOUFe) and registered at ClinicalTrials.gov (NCT07058896). All procedures were conducted in accordance with the Declaration of Helsinki and institutional ethical standards. Written informed consent was obtained from all participants before participation in the study. Participants provided consent for participation in the focus groups and prototype testing, as well as for audio/video recording and the anonymous use of collected data for research purposes. Participation was voluntary, and participants were informed that they could decline to answer any question or withdraw from the study at any time without consequences for their clinical care or relationship with the rehabilitation team. To ensure privacy and confidentiality, all collected data were pseudonymized prior to analysis and handled in accordance with the General Data Protection Regulation (GDPR; Regulation [EU] 2016/679). Audio and video recordings were used exclusively for transcription and analysis purposes and were deleted after completion of the analytic process. No personally identifiable information is reported in this manuscript. Participants did not receive financial compensation for participation in the study. No identifiable participant information or recognizable facial images are included in the manuscript figures or supplementary materials.

## Results

### Overview

The final application represents the culmination of a rigorous, user-centered iterative design process spanning 6 sequential phases (Figure 4), integrating qualitative input from distinct focus groups. Each phase informed subsequent iterations, enabling systematic resolution of identified limitations through targeted architectural modifications in adherence to ISO 9241-210 principles for human-centered design.

**Figure 4:**
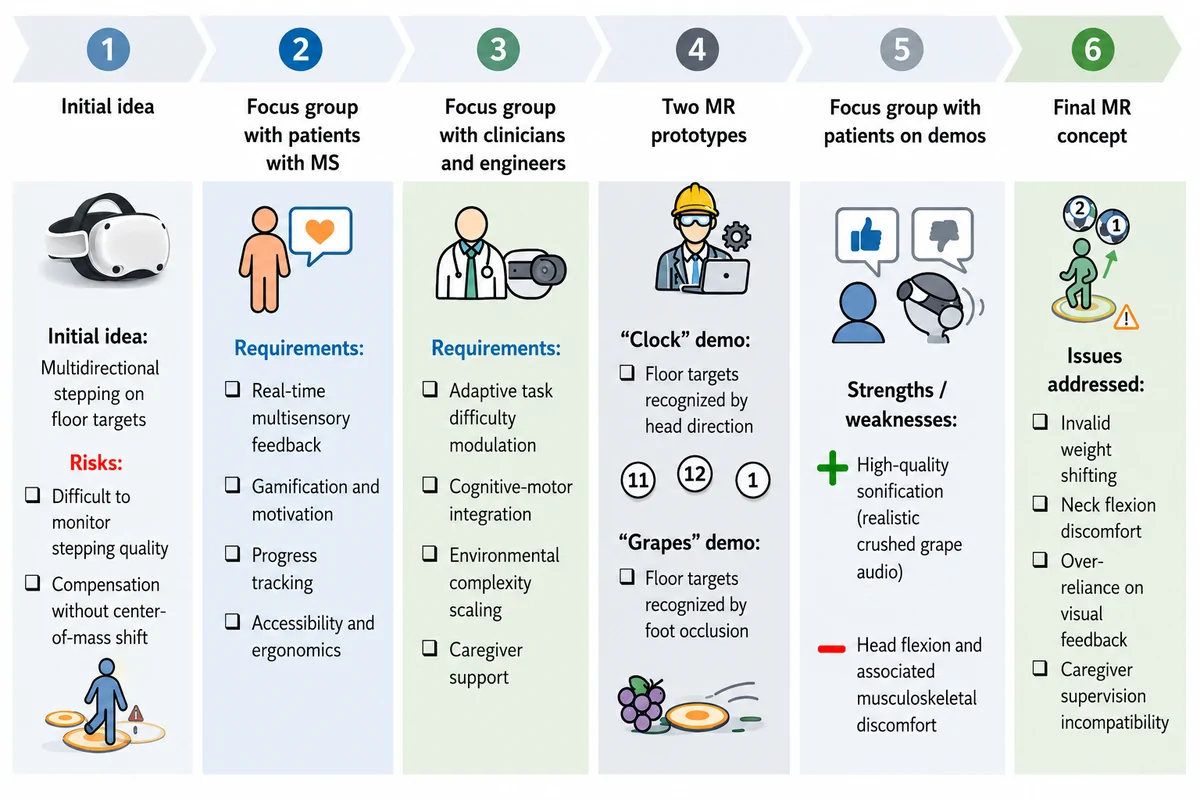
Co-design process of a mixed-reality (MR) balance-training application for people with multiple sclerosis (MS). The figure illustrates the iterative co-design and prototyping pathway used to develop the Head Your Limits MR application. The process comprised six sequential steps: (1) initial development of a rehabilitation concept based on multidirectional stepping on floor targets, highlighting key risks (difficulty in monitoring stepping quality and compensation without center-of-mass shift); (2) an online focus group with people with MS to elicit requirements related to real-time multisensory feedback, gamification and motivation, progress tracking, and accessibility/ergonomics; (3) an online focus group with clinicians and extended reality (XR) engineers to define requirements including adaptive task difficulty modulation, cognitive-motor integration, environmental complexity scaling, and caregiver support; (4) development of 2 MR prototypes (“clock” demo based on head-direction recognition and “grapes” demo based on foot-occlusion recognition); (5) in-person focus group with people with MS to discuss strengths and weaknesses of both demos (eg, high-quality sonification vs discomfort related to head flexion); and (6) final MR concept, addressing key issues (invalid weight shifting, neck flexion discomfort, over-reliance on visual feedback, and caregiver supervision incompatibility). Across all steps, subject, task, and environmental constraints were iteratively refined while the core motor skill—multidirectional stepping—remained unchanged.

### Phase 1: Conceptualization—Initial Idea

An initial application concept was formulated to address the persistent challenge of fall prevention in MS through volitional multidirectional step training. The proposed MR application implemented stepping response development using a Meta Quest 3 HMD running a Unity application presenting virtual floor-level targets arranged in a clock-face distribution. However, the task execution mechanism (foot occlusion of a virtual target) revealed a critical biomechanical limitation: patients could achieve foot-target contact while maintaining center-of-mass displacement on the stance limb, executing an isolated foot movement without genuine weight transfer. Additionally, floor-level targets required sustained downward head flexion, imposing postural burden and risk of compensatory strategies. The initial design lacked: (1) a biomechanical enforcement mechanism ensuring target achievement required valid weight transfer, and (2) ergonomic compatibility with neutral head posture and safe caregiver supervision. These unresolved problems motivated iterative refinement toward architectural modifications enforcing biomechanical validity while maintaining clinical and usability requirements.

### Phase 2: Patient-Centered Initial Requirements—Focus Group With Patients With MS

Fourteen people with MS attended an online focus group. The first patient focus group elicited user-derived requirements reflecting lived experience with motor rehabilitation and technology use preferences. Qualitative analysis identified five fundamental requirements: (1) closed-loop, real-time multisensory feedback enabling action-perception and motor error correction; (2) gamification and intrinsic motivation mechanisms sustaining long-term adherence; (3) personalized progress tracking fostering self-efficacy; (4) accessible visual design accommodating diverse visual abilities; and (5) ergonomic noninvasive design minimizing burden and compatible with corrective eyewear and mobility aids.

Patients reported an overall positive attitude toward the proposed MR application, describing curiosity, interest, and perceived usefulness. One participant felt that introducing this type of technology would be “a very positive step,” while others stated, “I am curious” and “I mainly see the positive aspects of the application; I am not afraid, and I do not perceive any particular risks.” Only a small number of participants reported initial apprehension, mainly related to being unfamiliar with the system or potential side effects. Participants emphasized the importance of balancing engagement with comfort and safety. Concerns were raised regarding possible nausea, sensory overload, and physical fatigue associated with headset use. For instance, one participant noted, “I might experience fatigue with the heavy headset,” while another commented that “the exercises seem quite tiring.” A further participant expressed concern about cybersickness, stating, “I heard that this type of application can make you feel sick, and this worries me a little.”

Participants expressed different views regarding sensory complexity and environmental distractions. Some considered additional stimuli potentially distracting or destabilizing, with one participant explaining that “the environment should be without distractions; too many stimuli would be worse for me.” Others, however, perceived increased sensory complexity as potentially motivating, provided that it was introduced progressively. Participants also highlighted the importance of clear multimodal feedback, expressing a preference for distinct auditory cues to signal task success or failure and for high-contrast visual elements accessible to users with visual impairments. Finally, the need for personalized training and individual performance reports was consistently mentioned as an important factor to support motivation and long-term engagement, with one participant stating that “personalized training would be perfect for me.”

### Phase 3: Clinical and Technical Requirements—Focus Group With Clinicians and Engineers

The interdisciplinary focus group established clinical specifications and identified technical constraints. Clinicians articulated positive design recommendations: adaptive task difficulty modulation, cognitive-motor integration through dual-task paradigms, and progressive environmental complexity scaling. As one participant noted, “increasing task difficulty would be optimal,” while another emphasized that “it would be important to add extra elements to increase the difficulty.” Critically, the following three design challenges were identified, requiring mitigation: (1) risk of visual overreliance potentially fostering proprioceptive underengagement and limiting functional transfer, (2) postural compensation from visual target pursuit inducing cervical burden and invalidating motor execution, and (3) safety risks requiring caregiver supervision while maintaining system accessibility.

Clinicians and engineers confirmed personalization as a major strength of the MR concept and supported adaptive modulation of task difficulty. One participant stated that they were “particularly impressed by the personalization.” Suggestions focused on increasing task complexity through cognitive-motor integration and environmental challenges to better approximate real-world balance demands. Safety considerations featured prominently in the discussion, particularly regarding home-based use and the need for supervision in more impaired patients. As one participant explained, “home-based application would be more critical, and it would be useful to have a caregiver present during training.” Technical concerns included the accuracy of foot contact detection, limitations of sensor-based measurements, and the potential impact of headset weight and altered visual perspective on movement behavior. Participants suggested combining visual and auditory feedback and integrating complementary measurement approaches to support more robust assessment, with one participant noting that “a global assessment/measurement would be useful.”

### Phase 4: Prototype Development—2 MR Prototypes

Phase 4 operationalized requirements and design challenges into 2 distinct prototypes exploring alternative solutions. The clock application prioritized ergonomic comfort through acoustic guidance, eliminating visual downward head flexion requirements. The grape application prioritized engagement through high-quality gamification (vineyard themes, realistic goal celebration and failure sounds, and ball trajectory animation). This parallel approach enabled comparative evaluation of design trade-offs.

### Phase 5: Comparative Usability Evaluation—Focus Group With Patients on Prototype Demos

The third focus group conducted hands-on comparative testing. The clock application demonstrated strong usability for acoustic guidance, reducing cervical burden and head flexion requirements. The grape application excelled in gamification quality and sonification realism, generating higher engagement. However, the grape application revealed a critical limitation: continuous visual field maintenance for floor-level targets required sustained head flexion, resulting in excessive cervical discomfort and increased vestibular load—empirically validating clinicians’ concerns about the risk of postural compensation. As an exploratory quantitative usability measure, the overall SUS score after hands-on testing indicated excellent perceived usability, with a median score of 95 (IQR 82.5-97.5). The SUS was administered once after the full hands-on testing session, as this phase was intended to provide a preliminary indication of the perceived usability of the overall MR training experience [50].

From the brief focus group discussion, participants reported that both MR demos were generally engaging and enjoyable, with initial apprehension related to the headset or task demands resolving quickly. Some participants described the headset experience as “really impressive” and “fascinating,” highlighting the novelty and positive impact of the immersive interaction. No symptoms of nausea or cybersickness were reported. The clock application was perceived as calmer and more comfortable due to its reliance on auditory guidance, which reduced the need for downward gaze. In contrast, the grape application was described as more dynamic and engaging, with participants commenting that “the game was very beautiful” and “very fun,” and appreciating the realistic and pleasant sound feedback. However, interaction with floor-level targets was associated with postural discomfort and reduced stability. One participant observed, “my posture is a bit strange,” while another reported that “when looking down, I lose my balance a little.” Participants therefore suggested presenting targets at a higher level to reduce head flexion. Additional observations included difficulties related to visual contrast and the use of corrective eyewear.

### Phase 6: Final MR Concept—Head Your Limits App

Phase 6 synthesized requirements and insights into the final application architecture, resolving identified tensions through integrated problem-solving. The core innovation repositioned targets from floor level to eye level (12-hour clock configuration) while shifting from foot-based occlusion to HMD position tracking. This modification simultaneously resolved multiple constraints reported in Table 1 and considered the following elements. First, ergonomic resolution: eye-level positioning enabled a neutral head posture, eliminating downward flexion burden, cervical strain, and vestibular load. Second, biomechanical validity: HMD tracking created kinematic coupling between target engagement and center-of-mass displacement onto the stepping limb, physically enforcing intended postural control. Third, proprioceptive preservation: distributed clock layout reduced visual guidance dependence on lower-limb movement, preserving proprioceptive engagement and reducing visual-dominance compensatory strategies. Fourth, caregiver proximity: HMD-based tracking permitted caregiver proximity without target occlusion, enabling physical support during execution. Fifth, gamification integration: realistic goal-celebration and crowd-disappointment sonification, with a soccer metaphor (ball-heading game), enhanced intrinsic motivation. Sixth, progress tracking: personalized end-of-session reports quantified successful contacts, distance, and balance quality, supporting self-efficacy. Seventh, cognitive-motor integration: eye-level distractors supported the training of rapid decision-making abilities.

The Head Your Limits application exemplifies unified problem-solving, in which ergonomic optimization, biomechanical validity, proprioceptive preservation, safety supervision, gamification, and progress tracking were integrated into a coherent architecture. Each modification reinforces the others: eye-level positioning enables ergonomic comfort and HMD tracking; HMD tracking enables caregiver proximity and proprioceptive preservation. The design directly addresses all clinician-identified challenges and integrates all patient-derived requirements, demonstrating how rigorous stakeholder engagement and iterative prototyping transform competing design tensions into integrated solutions aligned with therapeutic efficacy, patient experience, and clinical safety.

**Table 1:** Summary of stakeholder-derived requirements identified during the user-centered co-design process of the Head Your Limits application. The table summarizes key patient- and clinician-derived needs emerging across the co-design phases, their source, their translation into specific design solutions implemented in the final mixed-reality application, and the expected clinical benefit in people with MS^a^.

Problem or requirement	FG^b^ source	“Head Your Limits” application solution	Clinical benefit
Overreliance on visual feedback (proprioceptive loss)	Clinicians (FG2)	Neutral head posture reduces visual guidance on lower limbs; HMD^c^ tracking forces center-of-mass displacement	Preserved proprioceptive integration and reduced avoidance of weight shifting on the stepping leg
Neck flexion discomfort and vestibular overload	Patients (FG3, grape app)	Eye-level targets (no downward head flexion)	Eliminated cervical burden and sustained comfort for extended sessions
Lack of gamification and engagement	Patients (FG1)	Realistic goal celebration (audio + visual) vs crowd disappointment	Enhanced intrinsic motivation and higher session adherence
Insufficient progress tracking	Patients (FG1)	Personalized end-of-session reports (targets, distance, and balance quality)	Performance awareness and self-efficacy enhancement
Safety: caregiver supervision requirement	Clinicians (FG2)	HMD-based tracking (no hand and foot occlusion) allows caregiver proximity	Supervision feasible without interference and fall risk mitigation
Biomechanically invalid motor execution	Clinicians (FG2)	Head-actuated target requires center-of-mass shift onto stepping limb	Valid gait motor learning and functional transfer to real-world
Accessibility: head flexion during visual guidance	Clinicians (FG2)	12-hour clock layout at eye level	Distributed targets reduce sustained head flexion and inclusive design
Difficulty increase: progressive scaling of environmental complexity	Clinicians (FG2)	Eye-level target distractors	Training of rapid decision-making abilities

^a^MS: multiple sclerosis.

^b^FG: focus group.

^c^HMD: head-mounted display.

The application was named Head Your Limits to reflect its underlying rehabilitative rationale. The term head refers both to the act of heading a ball and, more broadly, to the active and intentional engagement required to challenge balance control. The word *limits* alludes to the participants’ limits of dynamic stability that the intervention aims to explore and expand. The name also conveys the idea that functional limitations are not solely determined by neurological impairment but are often shaped by conservative movement strategies adopted in response to fear of falling [53] and kinesiophobia [54]. In this sense, the application is designed to encourage users to safely challenge their limits, promoting exploratory and adaptive motor behavior. This perspective aligns with contemporary approaches in neurological rehabilitation, which emphasize the guided discovery of motor abilities and strategies rather than the mere repetition of predefined movement patterns [55].

## Discussion

### Principal Results

This study aimed to illustrate the user-centered iterative co-design process of a home-based MR rehabilitation application for volitional stepping training in people with MS. Our results show that integrating patient, clinician, and engineering perspectives enabled the identification and translation of key design requirements into the final Head Your Limits application while addressing common tensions in rehabilitation technology development, such as ergonomic burden, biomechanical validity, sensory reliance, caregiver involvement, and user engagement.

The adopted co-design methodology can be interpreted as a concrete operationalization of the 6 ISO 9241-210 principles reported by Ortiz-Escobar et al [46] in the context of assistive technology design. First, the principle that “the design is based upon an explicit understanding of users, tasks, and environments” was addressed by exploring the rehabilitation experiences, functional limitations, perceptual needs, safety concerns, and home-use constraints of people with MS, together with the clinical and technical requirements identified by rehabilitation professionals and an engineer. Second, the principle that “users are involved throughout design and development” was reflected in the sequential involvement of people with MS during the initial online focus groups and subsequent hands-on prototype testing [54], with clinician and engineering input incorporated between these patient-centered phases. Third, the principle that “the design is driven and refined by user-centered evaluation” was operationalized by using stakeholder feedback to develop, compare, and refine the 2 MR prototypes, with the strengths and limitations of the clock and grapes demos informing subsequent design decisions. Fourth, the iterative nature of the process was ensured by documenting the outputs of each phase, sharing them within the research team, and using them to guide the next development step. Fifth, the principle that “the design addresses the whole user experience” was reflected in the fact that evaluation was not limited to task performance but also considered comfort, fatigue, sensory load, visual accessibility, motivation, perceived safety, headset ergonomics, and caregiver compatibility. Finally, the principle that “the design team includes multidisciplinary skills and perspectives” was implemented by integrating the views of people with MS, physiotherapists, a physiatrist, rehabilitation researchers, and biomedical engineers. In this sense, ISO 9241-210 did not merely provide a conceptual reference for the study but functioned as an organizing framework linking stakeholder involvement, data collection, prototype comparison, iterative refinement, and final design decisions [46].

The co-design sessions culminated in the final Head Your Limits application, an MR-based SGs. In the game, players must hit different targets generated at eye level in a clock-based spatial layout with their heads, simulating a traditional soccer task. The transition from floor-level to eye-level targets combined with HMD-based tracking emerged as a pivotal design decision that enabled biomechanically valid task execution, easier integration of visual distractors, improved safety, and enhanced experiential quality within a single interaction paradigm. Although head-mediated interaction is commonly used in commercial VR entertainment and fitness games [56], its use in rehabilitation contexts has largely focused on engagement rather than on enforcing biomechanically valid movement strategies [57]. In the present application, head-based interaction was deliberately repurposed as a therapeutic constraint to promote center-of-mass displacement during volitional stepping [58], rather than as a purely game-driven interaction mechanic.

### Comparison With Prior Work

Previous studies in people with MS [20-22] have explored XR applications for gait adaptability, upper-limb rehabilitation, and personalized exergame-based gait training. Although recent studies on digital interventions for people with MS [59,60] have emphasized the value of participatory development, structured co-design approaches specifically focused on the development of MR-based SGs remain limited [22]. To the best of our knowledge, this is the first study to describe the development of an MR application for volitional step training in people with MS through a cross-sectional qualitative, user-centered co-design process. This study confirms that the participatory approach is particularly relevant for aligning technological functionalities with user needs and supporting the ecological relevance and long-term engagement of digital rehabilitation tools for people with MS [60,61].

Consistent with previous studies [62], the present findings support the inclusion of game-based elements as a relevant design components of MR-based rehabilitation applications. Specifically, participants identified game elements as important features to sustain interest and increase motivation and challenge during repeated training. They also highlighted the importance of clear visual and auditory feedback, including information on progress within the game. Progress tracking was primarily framed by participants as a motivational and self-monitoring feature, intended to support personal goal setting and self-efficacy during training. This is consistent with previous work [20-22,62] showing that feedback systems can support the training performance of people with MS and facilitate interaction with SGs. According to previous studies [20,21], another important design element that should be considered is the possibility of adapting the game based on user performance. This capacity for tailored task challenge adaptation may enable the delivery of this challenging stepping exercise even in people with MS with higher levels of disability, who represent a critical yet often overlooked target population for fall-prevention interventions [10]. Clinicians and engineers emphasized the importance of including a cognitive task during motor training. This is supported by existing evidence showing that dual-task training maximizes improvements in balance and mobility in people with MS [63] and promotes gains in both motor and cognitive outcomes [14].

Compared with existing technological solutions for volitional step training, Head Your Limits leverages the potential of MR to overcome the hardware constraints typically required to train stepping responses, such as stepping mats or exergame pads [14,16]. This flexibility is crucial to increase the number and spatial distribution of targets available to the participants, thereby enhancing both the ecological validity of the task and its individual tailoring to each participant’s postural control capacity [64]. Moreover, Head Your Limits presents interaction targets at eye level rather than at foot level. This design choice emerged from the co-design process and prototype testing, where floor-level targets were associated with downward gaze, cervical discomfort, and increased visual dependence on foot placement. By moving the targets to eye level, the final application preserves the therapeutic goal of volitional multidirectional stepping while encouraging body weight transfer and the need for continuous visual monitoring of the feet. This enables the prevention of compensatory mechanisms reported in people with MS, such as inappropriate weight transfer [65] and visual dependency while moving [66].

### Limitations

Some methodological limitations should be acknowledged. First, as is inherent in focus group methodology, some participants’ feedback may have been influenced by novelty effects, as some were new to MR technologies. Second, given the exploratory nature of focus groups involving early-stage concepts and prototypes, the ability to anticipate long-term usability, fatigue, or safety issues may be limited. Third, focus group dynamics may have influenced participants’ individual responses, thereby shaping the expression of personal experiences and needs. Furthermore, formal thematic saturation was not used as a criterion for participant recruitment or study completion. Consistent with the co-design nature of the study, participant involvement was guided by the inclusion of key stakeholder groups and the iterative needs of the development process rather than by a predefined saturation threshold. Consequently, although recurring themes emerged across focus groups, additional stakeholder input may have identified further requirements, preferences, or usability considerations relevant to the development of the mixed-reality application. Finally, the limited focus group sample size and our purposive recruitment strategy limit the representativeness and generalizability of our findings.

### Conclusions

In conclusion, this study demonstrates how iterative co-design methodology can effectively guide the development of an MR rehabilitation application by translating diverse stakeholder needs into coherent and clinically meaningful design solutions.

Building on the insights gained through this co-design process, several directions for the future development of the Head Your Limits application can be identified. In particular, by leveraging the adaptive capabilities of MR-based SG, task challenge progression can be personalized to individual motor abilities by dynamically adjusting the spatial and temporal characteristics of the stepping targets. Following a baseline assessment to identify each participant’s stepping limits, targets can be positioned to slightly exceed these limits, thereby promoting exploratory and adaptive stepping.

Although the present work does not evaluate clinical effectiveness, it provides a transparent and methodologically grounded account of the design process underpinning Head Your Limits, offering a replicable framework for the development of future rehabilitation technologies. Further work will be required to evaluate the usability, safety, and clinical impact of the MR application in individuals with MS, with effectiveness assessed through outcomes based on stepping performance and fall-related measures.

## Appendix

Multimedia Appendix 1 Discussion guide for the patient focus group.

Multimedia Appendix 2 Discussion guide for the focus group with clinicians and engineers.

Multimedia Appendix 3 Focus group questions administered during the third in-person patient focus group.

## Abbreviations

AVEC: Ethics Committee Area Vasta Emilia Centro
EDSS: Expanded Disability Status Scale
GDPR: General Data Protection Regulation
HMD: head-mounted display
ISO: International Organization for Standardization
MMSE: Mini-Mental State Examination
MR: mixed reality
MS: multiple sclerosis
SG: serious game
SUS: System Usability Scale
XR: extended reality
