# Supplementary material for: Development of a Mixed-Reality Application for Volitional Step Training in People With Multiple Sclerosis: Cross-Sectional Qualitative User-Centered Co-Design Study
Source: JMIR Serious Games. 2026 Sep 14;14:e94312. doi: 10.2196/94312 (PMC13620433; doi:10.2196/94312)
Supplement: Supplementary file 1 [file games_v14i1e94312_app1.docx]

### Appendix 1.a. Discussion guide for the patient focus group

Focus group type: Semi-structured

Aim: to explore the perspectives of individuals with MS regarding their rehabilitation experiences, expectations, preferences, and perceived acceptability of mixed reality-based training

Participants: Patients with Multiple Sclerosis

Moderators: two trained researchers

Duration: 60 minutes

1.	**Introduction**

- Welcome and explanation of the study aims.
- Explanation of the focus group format and voluntary participation
- Assurance of confidentiality and anonymized data handling
- Brief round of introductions

*Guiding question*:

- What is the first word that comes to your mind when you think about technology in rehabilitation?

2.	**Warm-up, experiences and expectations**

*Guiding questions*:

- What do you expect from technology designed to help improve your safety during movement?
- Is there anything that particularly interests or concerns you about this type of technology?
- At what moments during the day do you feel most unstable?
- Have you ever tried other technological tools aimed at improving balance?

3.	**Brief explanation of the mixed reality concept** (initial idea of multidirectional stepping on floor targets)

4. **Reactions and open discussion**

Guiding questions:

- What aspects of this system seem most useful, and which appear more complex or difficult?
- Would you feel comfortable using this tool?

5. **Preferences, personalization, and feedback**

*Guiding questions*:

- Would you like to receive immediate feedback (e.g., visual or auditory signals)?
- Which type of feedback would you prefer: auditory, visual, or both?
- Would you like the system to be customizable according to your needs?
- What form should the target to be reached with the foot have?
- For how long do you think you could perform this task continuously?
- What elements would help you maintain attention during the task over time?

6. **Emotions and perceived safety**

*Guiding questions*:

- How do you think using this tool would make you feel?
- Would you feel safe performing this mixed reality exercise at home?
- Do you think it would help you feel more confident during movement?

1. **Closing**

*Guiding questions:*

- Is there anything else you would like to add?
- Is there anything you think should be avoided?
- How was your experience of participating today?
