# Supplementary material for: Development of a Mixed-Reality Application for Volitional Step Training in People With Multiple Sclerosis: Cross-Sectional Qualitative User-Centered Co-Design Study
Source: JMIR Serious Games. 2026 Sep 14;14:e94312. doi: 10.2196/94312 (PMC13620433; doi:10.2196/94312)
Supplement: Supplementary file 2 [file games_v14i1e94312_app2.docx]

### Appendix 1.b. Discussion guide for the Focus Group with Clinicians & Engineers

Focus group type: Semi-structured

Aim: to gather interdisciplinary input from healthcare professionals and an engineer to contextualize patient perspectives within clinical and technical constraints

Participants: Healthcare professionals (n = 4 clinicians) and one engineer

Moderators: Three trained researchers

Duration: Approximately 60 minutes

1.	**Introduction**

- Welcome and explanation of the study aims
- Explanation of the focus group format and voluntary participation
- Assurance of confidentiality and anonymized data handling
- Brief round of introductions, including professional role and experience with innovative technologies

*Guiding question*:

- Could you briefly introduce your role and your experience with innovative technologies in clinical, rehabilitative, or technological contexts?

2.	**Current experiences and unmet needs**

*Guiding questions:*

- From a clinical perspective, which motor rehabilitation needs related to mobility and postural control remain insufficiently addressed in neurological conditions?
- From a technological perspective, which challenges are most encountered when developing mixed reality systems for clinical contexts?

3.	**Presentation of the mixed reality system**

- Brief presentation of the mixed reality prototype.
- Explanation of the intended clinical and technical rationale.

4.	**Initial reactions and open discussion**

*Guiding questions*:

- What aspects of the proposed system appear most promising, and which raise concerns or uncertainties?
- Which elements do you consider particularly relevant, considering patients’ needs for clear feedback, well-distinguishable visual elements, brief exercises, and motivational reports?

5.	**Objectives, monitoring, and feedback**

*Guiding questions:*

- Which rehabilitation objectives do you consider realistically achievable using a mixed reality system?
- For which patient profiles would this system be most appropriate?
- Which parameters or metrics should be prioritized to ensure clinical usefulness, and which technical constraints should be considered when designing simple and clear visual or auditory feedback?
- Which types of feedback would be useful for patients, and which might be counterproductive?
- Which aspects of system adaptability (e.g., variation in target distance, speed, or size) are technically simpler or more complex to implement?

6.	**Implementation, safety, and sustainability**

*Guiding questions*:

- At which stage of the rehabilitation pathway would you integrate a mixed reality system?
- Would you consider autonomous use at home appropriate, and for which patient profiles?
- Which minimum technical requirements are necessary to ensure safety and reliability?
- Which infrastructural or maintenance aspects are critical for long-term sustainability?

7.	**Closing**

*Guiding questions:*

- What do you consider the priority recommendations for improving this system?
- Which clinical or technical risks should be carefully prevented?
