# Supplementary material for: Development of a Mixed-Reality Application for Volitional Step Training in People With Multiple Sclerosis: Cross-Sectional Qualitative User-Centered Co-Design Study
Source: JMIR Serious Games. 2026 Sep 14;14:e94312. doi: 10.2196/94312 (PMC13620433; doi:10.2196/94312)
Supplement: Supplementary file 3 [file games_v14i1e94312_app3.docx]

### Appendix 1.c. Focus group questions administered during the third in-person patient focus group

Focus group type: Semi-structured with hands-on system demonstration

Aim: to evaluate user interaction with the mixed reality application through hands-on testing

Participants: Patients with Multiple Sclerosis (n=9)

Moderators: Two trained researchers

Duration: Approximately 120 minutes

Open-ended questions for focus group discussion

- Which sound did you prefer (clock or grapes)?
- Which type of target did you prefer? (Why?)
- How did you feel in the two scenarios?
